# Bis(ferrocenecarbaldehyde 4-methyl­thio­semicarbazonato-κ^2^ *N* ^1^,*S*)zinc(II) methanol solvate

**DOI:** 10.1107/S1600536809030086

**Published:** 2009-08-08

**Authors:** M. R. Vikneswaran, Siang Guan Teoh, Ching Kheng Quah, Hoong-Kun Fun

**Affiliations:** a School of Chemical Sciences, Universiti Sains Malaysia, 11800 USM, Penang, Malaysia; b X-ray Crystallography Unit, School of Physics, Universiti Sains Malaysia, 11800 USM, Penang, Malaysia

## Abstract

In the title compound, [Fe_2_Zn(C_5_H_5_)_2_(C_8_H_9_N_3_S)_2_]·CH_3_OH, the dihedral angles between the substituted and unsubstituted cyclo­penta­dienyl rings are 89.34 (8) and 85.73 (9)°, respectively. The two Zn/S/C/N/N five-membered rings adopt envelope conformations, with the Zn^II^ atom at the flap. Each methanol solvent mol­ecule is linked to three ferrocene groups *via* inter­molecular O—H⋯N, N—H⋯O and C—H⋯O hydrogen bonds. The crystal structure is further consolidated by C—H⋯π inter­actions.

## Related literature

For related structures, see: Vikneswaran *et al.* (2009 ▶); Seiler & Dunitz (1979 ▶). For the preparation, see: Mariño *et al.* (2006 ▶). For ring conformations, see: Cremer & Pople (1975 ▶). For the stability of the temperature controller used for the data collection, see: Cosier & Glazer (1986 ▶).
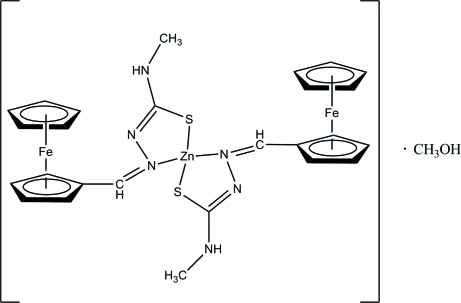


## Experimental

### 

#### Crystal data

[Fe_2_Zn(C_5_H_5_)_2_(C_8_H_9_N_3_S)_2_]·CH_4_O
*M* *_r_* = 697.78
Monoclinic, 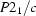

*a* = 10.9490 (1) Å
*b* = 9.4365 (1) Å
*c* = 28.1899 (3) Å
β = 101.448 (1)°
*V* = 2854.64 (5) Å^3^
*Z* = 4
Mo *K*α radiation
μ = 2.02 mm^−1^
*T* = 100 K
0.36 × 0.21 × 0.15 mm

#### Data collection

Bruker SMART APEXII CCD area-detector diffractometer
Absorption correction: multi-scan (**SADABS**; Bruker, 2005 ▶) *T*_min_ = 0.532, *T*_max_ = 0.752
66919 measured reflections
14925 independent reflections
11613 reflections with *I* > 2σ(*I*)
*R*_int_ = 0.036

#### Refinement

*R*[*F* ^2^ > 2σ(*F* ^2^)] = 0.034
*wR*(*F* ^2^) = 0.085
*S* = 1.03
14925 reflections
355 parameters
H-atom parameters constrained
Δρ_max_ = 1.63 e Å^−3^
Δρ_min_ = −0.33 e Å^−3^

### 

Data collection: *APEX2* (Bruker, 2005 ▶); cell refinement: *SAINT* (Bruker, 2005 ▶); data reduction: *SAINT*; program(s) used to solve structure: *SHELXTL* (Sheldrick, 2008 ▶); program(s) used to refine structure: *SHELXTL*; molecular graphics: *SHELXTL*; software used to prepare material for publication: *SHELXTL* and *PLATON* (Spek, 2009 ▶).

## Supplementary Material

Crystal structure: contains datablocks global, I. DOI: 10.1107/S1600536809030086/ci2857sup1.cif

Structure factors: contains datablocks I. DOI: 10.1107/S1600536809030086/ci2857Isup2.hkl

Additional supplementary materials:  crystallographic information; 3D view; checkCIF report

## Figures and Tables

**Table 1:** Hydrogen-bond geometry (Å, °)

*D*—H⋯*A*	*D*—H	H⋯*A*	*D*⋯*A*	*D*—H⋯*A*
O1—H1⋯N4^i^	0.82	2.13	2.9460 (16)	178
N5—H2⋯O1^ii^	0.84	2.06	2.8714 (17)	162
C18—H18*A*⋯O1^iii^	0.98	2.49	3.4065 (19)	156
C1—H1*A*⋯*Cg*3^iv^	0.98	2.84	3.6176 (15)	137
C11—H11*A*⋯*Cg*1^v^	0.98	2.58	3.4280 (15)	145
C12—H12*A*⋯*Cg*5^iii^	0.98	2.68	3.6607 (14)	175
C27—H27*A*⋯*Cg*4^i^	0.96	2.79	3.3830 (18)	121

Symmetry codes: (i) 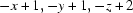; (ii) 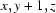; (iii) 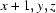; (iv) 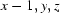; (v) 
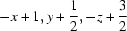
. *Cg*1, *Cg*3, *Cg*4 and *Cg*5 are the centroids of the C1–C5, C11–C15, C16–C20 and Zn1/S1/C22/N2/N1 rings, respectively.

**Fig. 1.**
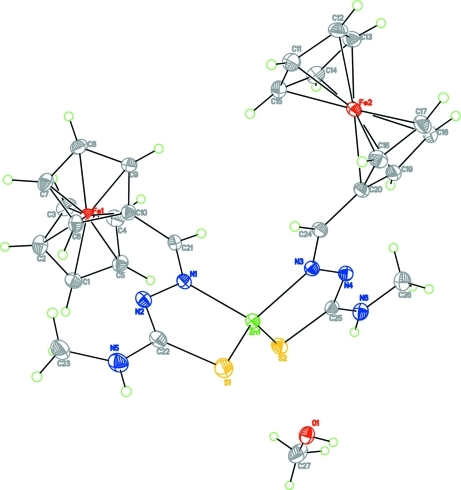
The molecular structure of the title compound, showing 50% probability displacement ellipsoids and the atom-numbering scheme.

**Fig. 2.**
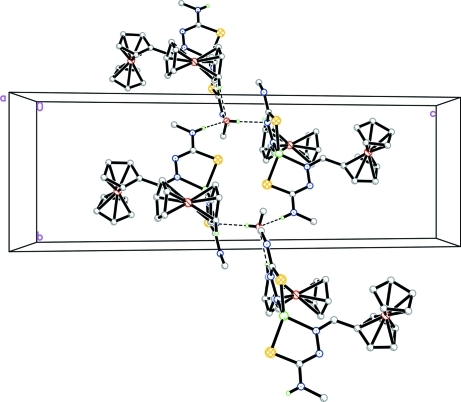
Part of the crystal packing of the title compound, viewed along the a axis. H atoms not involved in hydrogen bonds (dashed lines) have been omitted for clarity.

## supplementary crystallographic information

### Comment

As a continuation of our research related to ferrocenyl thiosemicarbazones and its metal complexes, herein we report the synthesis and crystal structure of a Zn^II^ complex formed with formylferrocene 4-methylthiosemicarbazone.

The Fe—C distances (Fig. 1) are as expected for a ferrocene derivative, and are comparable to those observed in closely related structures (Seiler & Dunitz, 1979; Vikneswaran *et al.*, 2009). In the ferrocene groups, the Fe1···*Cg*1/*Cg*2 and Fe2···*Cg*3/*Cg*4 distances are 1.6514 (7)/1.6453 (6) Å, and 1.6564 (6)/1.6497 (6) Å, respectively, and the *Cg*···Fe···*Cg* angle is 178.75 (3) and 175.27 (3)°, respectively for Fe1 and Fe2 atoms, where *Cg*1, *Cg*3 and *Cg*2, *Cg*4 are the centroids of the unsubstituted [Cp1 (C1—C5) and Cp3 (C11—C15)] and substituted [Cp2 (C6—C10) and Cp4 (C16—C20)] Cp rings, respectively. The dihedral angle between the substituted Cp rings is 89.34 (8)° [the value for the unsubstituted Cp rings = 85.73 (9)°] indicating that the two ferrocene groups position themselves almost perpendicular to each other. Each of the Cp1/Cp2 and Cp3/Cp4 pairs are approximately parallel [dihedral angle for Cp1/Cp2 = 2.05 (8) and for Cp3/Cp4 = 5.73 (8)°] and each ring is essentially planar [maximum deviation for Cp1 is 0.002 (2) Å for atom C2; for Cp2 is 0.007 (1) Å for atom C9; for Cp3 is 0.002 (1) Å for atoms C12, C13 and C14; and for Cp4 is 0.010 (1) Å for atom C16].

The angles around the Zn1 atom ranges from 86.60 (3) to 132.032 (14)°, forming a distorted tetrahedral environment. The two five-membered rings, Zn1/S1/C22/N2/N1 (A) and Zn1/S2/C25/N4/N3 (B), adopt envelope conformations with the Zn atom at the flap; the puckering parameters (Cremer & Pople, 1975) are Q = 0.1507 (8) Å and Θ = 187.6 (5)° for ring A, and Q = 0.1111 (8) Å and Θ = 177.9 (6)° for ring B. In the solid state (Fig 2), each methanol molecule is linked to three ferrocene molecules *via* intermolecular O1—H1···N4, N5—H2···O1 and C18—H18A···O1 hydrogen bonds. The crystal structure is further consolidated by C—H···π interactions (Table 1).

### Experimental

Formylferrocene 4-methylthiosemicarbazone was prepared as described by Mariño *et al.* (2006). Zn(CH_3_COO)_2_.2H_2_O (0.21 g, 1 mmol) dissolved in methanol (60 ml) was added dropwise at room temperature to a mixture of formylferrocene 4-methylthiosemicarbazone (0.30 g, 1 mmol) and KOH (0.12 g, 2 mmol) in absolute methanol (15 ml). Amorphous orange solids separated out immediately. The suspension was stirred under reflux for 4 h and filtered. After several days, brown crystals were obtained from the filtrate.

### Refinement

N-bound and O-bound H atoms were located in a difference Fourier map and refined as riding with the parent atom with an isotropic thermal parameter 1.2 and 1.5 times, respectively, that of the parent atom. All other H atoms were placed in calculated positions, with C–H = 0.93–0.98 Å, and refined using a riding model, with *U*_iso_(H) = 1.2 or 1.5 *U*_eq_(C). A rotating-group model was applied for the methyl groups. The maximum and minimum residual electron density peaks of 1.63 and -0.33 eÅ^-3^, respectively, were located at 0.80 Å and 0.36 Å from the Fe2 and H27B atoms, respectively.

### Crystal data

**Table:**

[Fe_2_Zn(C_5_H_5_)_2_(C_8_H_9_N_3_S)_2_]·CH_4_O	*F*(000) = 1432
*M**_r_* = 697.78	*D*_x_ = 1.624 Mg m^−^^3^
Monoclinic, *P*2_1_/*c*	Mo *K*α radiation, λ = 0.71073 Å
Hall symbol: -P 2ybc	Cell parameters from 9581 reflections
*a* = 10.9490 (1) Å	θ = 2.3–39.8°
*b* = 9.4365 (1) Å	µ = 2.02 mm^−^^1^
*c* = 28.1899 (3) Å	*T* = 100 K
β = 101.448 (1)°	Block, brown
*V* = 2854.64 (5) Å^3^	0.36 × 0.21 × 0.15 mm
*Z* = 4	

### Data collection

**Table:**

Bruker SMART APEXII CCD area-detector diffractometer	14925 independent reflections
Radiation source: fine-focus sealed tube	11613 reflections with *I* > 2σ(*I*)
graphite	*R*_int_ = 0.036
φ and ω scans	θ_max_ = 37.5°, θ_min_ = 1.5°
Absorption correction: multi-scan (*SADABS*; Bruker, 2005)	*h* = −18→18
*T*_min_ = 0.532, *T*_max_ = 0.752	*k* = −15→15
66919 measured reflections	*l* = −48→48

### Refinement

**Table:**

Refinement on *F*^2^	Primary atom site location: structure-invariant direct methods
Least-squares matrix: full	Secondary atom site location: difference Fourier map
*R*[*F*^2^ > 2σ(*F*^2^)] = 0.034	Hydrogen site location: inferred from neighbouring sites
*wR*(*F*^2^) = 0.085	H-atom parameters constrained
*S* = 1.03	*w* = 1/[σ^2^(*F*_o_^2^) + (0.0372*P*)^2^ + 1.1076*P*] where *P* = (*F*_o_^2^ + 2*F*_c_^2^)/3
14925 reflections	(Δ/σ)_max_ = 0.004
355 parameters	Δρ_max_ = 1.63 e Å^−^^3^
0 restraints	Δρ_min_ = −0.33 e Å^−^^3^

### Special details

**Table:**

Experimental. The crystal was placed in the cold stream of an Oxford Cyrosystems Cobra open-flow nitrogen cryostat (Cosier & Glazer, 1986) operating at 100.0 (1) K.
Geometry. All e.s.d.'s (except the e.s.d. in the dihedral angle between two l.s. planes) are estimated using the full covariance matrix. The cell e.s.d.'s are taken into account individually in the estimation of e.s.d.'s in distances, angles and torsion angles; correlations between e.s.d.'s in cell parameters are only used when they are defined by crystal symmetry. An approximate (isotropic) treatment of cell e.s.d.'s is used for estimating e.s.d.'s involving l.s. planes.
Refinement. Refinement of *F*^2^ against ALL reflections. The weighted *R*-factor *wR* and goodness of fit *S* are based on *F*^2^, conventional *R*-factors *R* are based on *F*, with *F* set to zero for negative *F*^2^. The threshold expression of *F*^2^ > σ(*F*^2^) is used only for calculating *R*-factors(gt) *etc*. and is not relevant to the choice of reflections for refinement. *R*-factors based on *F*^2^ are statistically about twice as large as those based on *F*, and *R*- factors based on ALL data will be even larger.

### Fractional atomic coordinates and isotropic or equivalent isotropic displacement parameters (Å^2^)

**Table:**

	*x*	*y*	*z*	*U*_iso_*/*U*_eq_	
Zn1	0.466846 (13)	0.874062 (18)	0.913577 (5)	0.01586 (3)	
Fe1	0.306836 (16)	0.86548 (2)	0.704222 (6)	0.01419 (4)	
Fe2	0.985568 (15)	0.80244 (2)	0.880148 (6)	0.01416 (4)	
S1	0.41083 (3)	1.08484 (4)	0.941830 (12)	0.02241 (7)	
S2	0.39805 (3)	0.64888 (4)	0.920651 (13)	0.02035 (6)	
N1	0.43015 (9)	0.96457 (12)	0.84659 (4)	0.01548 (18)	
N2	0.36985 (9)	1.09421 (13)	0.84119 (4)	0.01673 (19)	
N3	0.64175 (9)	0.79164 (12)	0.92541 (4)	0.01518 (18)	
N4	0.65645 (9)	0.65047 (12)	0.93923 (4)	0.01542 (18)	
N5	0.29437 (11)	1.27509 (14)	0.88056 (5)	0.0223 (2)	
H2	0.2801	1.3094	0.9066	0.027*	
N6	0.55829 (10)	0.44289 (13)	0.94845 (4)	0.0196 (2)	
H3	0.4950	0.3975	0.9494	0.024*	
C1	0.13526 (12)	0.80316 (16)	0.71554 (5)	0.0201 (2)	
H1A	0.0836	0.8543	0.7345	0.024*	
C2	0.13267 (13)	0.82038 (16)	0.66519 (5)	0.0221 (2)	
H2A	0.0787	0.8855	0.6434	0.027*	
C3	0.22260 (14)	0.72664 (17)	0.65208 (5)	0.0247 (3)	
H3A	0.2412	0.7160	0.6197	0.030*	
C4	0.28126 (13)	0.65217 (16)	0.69439 (6)	0.0232 (3)	
H4A	0.3472	0.5809	0.6962	0.028*	
C5	0.22705 (12)	0.69916 (15)	0.73344 (5)	0.0206 (2)	
H5A	0.2496	0.6663	0.7670	0.025*	
C6	0.34723 (11)	1.06143 (14)	0.73498 (5)	0.0173 (2)	
H6A	0.2916	1.1198	0.7502	0.021*	
C7	0.35675 (12)	1.06461 (15)	0.68519 (5)	0.0195 (2)	
H7A	0.3079	1.1251	0.6601	0.023*	
C8	0.44765 (12)	0.96267 (16)	0.67786 (5)	0.0203 (2)	
H8A	0.4720	0.9415	0.6470	0.024*	
C9	0.49386 (11)	0.89433 (16)	0.72265 (5)	0.0184 (2)	
H9A	0.5569	0.8193	0.7283	0.022*	
C10	0.43300 (11)	0.95626 (14)	0.75871 (4)	0.0159 (2)	
C11	0.98425 (12)	0.94343 (15)	0.82424 (5)	0.0207 (2)	
H11A	0.9689	1.0456	0.8252	0.025*	
C12	1.10230 (12)	0.87512 (16)	0.83719 (5)	0.0213 (2)	
H12A	1.1826	0.9223	0.8484	0.026*	
C13	1.08336 (12)	0.72681 (16)	0.83113 (5)	0.0212 (2)	
H13A	1.1483	0.6539	0.8373	0.025*	
C14	0.95335 (13)	0.70258 (16)	0.81409 (5)	0.0204 (2)	
H14A	0.9132	0.6102	0.8067	0.024*	
C15	0.89247 (12)	0.83665 (16)	0.81002 (5)	0.0196 (2)	
H15A	0.8027	0.8525	0.7997	0.024*	
C16	0.97126 (11)	0.91423 (16)	0.94052 (5)	0.0199 (2)	
H16A	0.9637	1.0174	0.9427	0.024*	
C17	1.08492 (11)	0.83541 (18)	0.94877 (5)	0.0226 (3)	
H17A	1.1691	0.8752	0.9573	0.027*	
C18	1.05450 (12)	0.68873 (17)	0.94153 (5)	0.0216 (3)	
H18A	1.1145	0.6107	0.9440	0.026*	
C19	0.92240 (11)	0.67500 (15)	0.92959 (5)	0.0176 (2)	
H19A	0.8758	0.5863	0.9224	0.021*	
C20	0.86960 (10)	0.81478 (15)	0.92949 (4)	0.0160 (2)	
C21	0.45564 (11)	0.90477 (15)	0.80830 (4)	0.0162 (2)	
H21A	0.4938	0.8165	0.8133	0.019*	
C22	0.35672 (11)	1.15094 (15)	0.88276 (5)	0.0176 (2)	
C23	0.22804 (14)	1.33448 (18)	0.83546 (6)	0.0261 (3)	
H23A	0.1753	1.4102	0.8421	0.039*	
H23B	0.1780	1.2622	0.8170	0.039*	
H23C	0.2868	1.3705	0.8173	0.039*	
C24	0.74080 (11)	0.86180 (14)	0.92128 (4)	0.0160 (2)	
H24A	0.7275	0.9558	0.9117	0.019*	
C25	0.54956 (11)	0.58180 (15)	0.93734 (4)	0.0157 (2)	
C26	0.67565 (13)	0.36712 (16)	0.95984 (5)	0.0230 (3)	
H26A	0.6598	0.2673	0.9612	0.035*	
H26B	0.7219	0.3985	0.9906	0.035*	
H26C	0.7230	0.3854	0.9353	0.035*	
O1	0.19264 (10)	0.36504 (11)	0.96199 (4)	0.02148 (19)	
H1	0.2355	0.3592	0.9891	0.032*	
C27	0.09851 (15)	0.2593 (2)	0.95292 (6)	0.0336 (4)	
H27A	0.0293	0.2877	0.9670	0.050*	
H27B	0.1315	0.1713	0.9670	0.050*	
H27C	0.0711	0.2475	0.9186	0.050*	

### Atomic displacement parameters (Å^2^)

**Table:**

	*U*^11^	*U*^22^	*U*^33^	*U*^12^	*U*^13^	*U*^23^
Zn1	0.01284 (5)	0.01938 (8)	0.01524 (6)	0.00138 (5)	0.00252 (4)	0.00098 (6)
Fe1	0.01512 (7)	0.01382 (8)	0.01383 (7)	−0.00051 (6)	0.00337 (5)	0.00039 (6)
Fe2	0.01090 (6)	0.01444 (8)	0.01707 (7)	−0.00035 (6)	0.00264 (5)	−0.00001 (7)
S1	0.02330 (14)	0.02456 (17)	0.01853 (13)	0.00411 (13)	0.00209 (11)	−0.00436 (13)
S2	0.01366 (11)	0.02284 (16)	0.02418 (14)	−0.00291 (11)	0.00286 (10)	0.00307 (13)
N1	0.0129 (4)	0.0166 (5)	0.0169 (4)	0.0012 (3)	0.0028 (3)	0.0007 (4)
N2	0.0150 (4)	0.0153 (5)	0.0200 (4)	0.0017 (4)	0.0035 (3)	−0.0002 (4)
N3	0.0141 (4)	0.0158 (5)	0.0155 (4)	−0.0006 (3)	0.0028 (3)	−0.0001 (4)
N4	0.0148 (4)	0.0153 (5)	0.0159 (4)	−0.0008 (3)	0.0026 (3)	0.0005 (4)
N5	0.0239 (5)	0.0182 (5)	0.0257 (5)	0.0036 (4)	0.0071 (4)	−0.0025 (5)
N6	0.0200 (4)	0.0181 (5)	0.0201 (5)	−0.0036 (4)	0.0022 (4)	0.0025 (4)
C1	0.0173 (5)	0.0193 (6)	0.0243 (6)	−0.0025 (4)	0.0051 (4)	−0.0018 (5)
C2	0.0222 (5)	0.0199 (6)	0.0214 (6)	−0.0040 (5)	−0.0029 (4)	−0.0001 (5)
C3	0.0336 (7)	0.0205 (7)	0.0210 (6)	−0.0072 (6)	0.0078 (5)	−0.0057 (5)
C4	0.0243 (6)	0.0148 (6)	0.0316 (7)	−0.0007 (5)	0.0083 (5)	−0.0011 (5)
C5	0.0221 (5)	0.0179 (6)	0.0218 (5)	−0.0038 (5)	0.0045 (4)	0.0031 (5)
C6	0.0186 (5)	0.0140 (5)	0.0182 (5)	−0.0007 (4)	0.0009 (4)	0.0008 (4)
C7	0.0214 (5)	0.0175 (6)	0.0186 (5)	−0.0037 (4)	0.0015 (4)	0.0037 (5)
C8	0.0202 (5)	0.0244 (7)	0.0171 (5)	−0.0037 (5)	0.0056 (4)	0.0024 (5)
C9	0.0151 (4)	0.0231 (6)	0.0175 (5)	0.0003 (4)	0.0046 (4)	0.0013 (5)
C10	0.0157 (4)	0.0165 (6)	0.0153 (5)	−0.0003 (4)	0.0022 (4)	0.0005 (4)
C11	0.0216 (5)	0.0151 (6)	0.0260 (6)	0.0005 (4)	0.0064 (5)	0.0038 (5)
C12	0.0169 (5)	0.0224 (7)	0.0264 (6)	−0.0022 (5)	0.0088 (4)	0.0028 (5)
C13	0.0216 (5)	0.0193 (6)	0.0249 (6)	0.0040 (5)	0.0099 (5)	0.0019 (5)
C14	0.0249 (5)	0.0173 (6)	0.0193 (5)	−0.0020 (5)	0.0053 (4)	−0.0014 (5)
C15	0.0187 (5)	0.0206 (6)	0.0188 (5)	0.0009 (4)	0.0022 (4)	0.0025 (5)
C16	0.0161 (4)	0.0216 (6)	0.0215 (5)	−0.0030 (4)	0.0028 (4)	−0.0047 (5)
C17	0.0137 (4)	0.0316 (8)	0.0211 (5)	−0.0009 (5)	0.0003 (4)	−0.0034 (6)
C18	0.0160 (5)	0.0277 (7)	0.0205 (5)	0.0047 (5)	0.0019 (4)	0.0043 (5)
C19	0.0160 (4)	0.0181 (6)	0.0190 (5)	0.0015 (4)	0.0039 (4)	0.0027 (5)
C20	0.0127 (4)	0.0179 (6)	0.0174 (5)	−0.0001 (4)	0.0031 (4)	−0.0006 (4)
C21	0.0148 (4)	0.0168 (6)	0.0166 (5)	0.0020 (4)	0.0022 (4)	0.0019 (4)
C22	0.0144 (4)	0.0161 (6)	0.0222 (5)	−0.0002 (4)	0.0034 (4)	−0.0016 (5)
C23	0.0231 (6)	0.0235 (7)	0.0328 (7)	0.0071 (5)	0.0081 (5)	0.0038 (6)
C24	0.0141 (4)	0.0159 (5)	0.0182 (5)	0.0005 (4)	0.0038 (4)	0.0007 (4)
C25	0.0159 (4)	0.0182 (6)	0.0126 (4)	−0.0020 (4)	0.0022 (4)	0.0001 (4)
C26	0.0259 (6)	0.0178 (6)	0.0243 (6)	0.0013 (5)	0.0027 (5)	−0.0004 (5)
O1	0.0251 (4)	0.0193 (5)	0.0188 (4)	−0.0008 (4)	0.0013 (3)	−0.0008 (4)
C27	0.0280 (6)	0.0505 (11)	0.0207 (6)	−0.0146 (7)	0.0007 (5)	−0.0007 (7)

### Geometric parameters (Å, °)

**Table:**

Zn1—N3	2.0322 (10)	C4—H4A	0.98
Zn1—N1	2.0385 (11)	C5—H5A	0.98
Zn1—S1	2.2716 (4)	C6—C7	1.4284 (18)
Zn1—S2	2.2764 (4)	C6—C10	1.4369 (18)
Fe1—C1	2.0529 (13)	C6—H6A	0.98
Fe1—C2	2.0482 (13)	C7—C8	1.428 (2)
Fe1—C3	2.0468 (15)	C7—H7A	0.98
Fe1—C4	2.0436 (15)	C8—C9	1.4186 (19)
Fe1—C5	2.0470 (14)	C8—H8A	0.98
Fe1—C6	2.0529 (14)	C9—C10	1.4449 (17)
Fe1—C7	2.0577 (14)	C9—H9A	0.98
Fe1—C8	2.0556 (13)	C10—C21	1.4540 (17)
Fe1—C9	2.0286 (12)	C11—C15	1.423 (2)
Fe1—C10	2.0394 (13)	C11—C12	1.4256 (19)
Fe2—C11	2.0603 (14)	C11—H11A	0.98
Fe2—C12	2.0457 (13)	C12—C13	1.420 (2)
Fe2—C13	2.0377 (13)	C12—H12A	0.98
Fe2—C14	2.0542 (14)	C13—C14	1.4276 (19)
Fe2—C15	2.0618 (13)	C13—H13A	0.98
Fe2—C16	2.0347 (14)	C14—C15	1.424 (2)
Fe2—C17	2.0462 (14)	C14—H14A	0.98
Fe2—C18	2.0486 (14)	C15—H15A	0.98
Fe2—C19	2.0614 (13)	C16—C17	1.4286 (19)
Fe2—C20	2.0645 (12)	C16—C20	1.4419 (18)
S1—C22	1.7668 (14)	C16—H16A	0.98
S2—C25	1.7504 (13)	C17—C18	1.429 (2)
N1—C21	1.2965 (16)	C17—H17A	0.98
N1—N2	1.3841 (16)	C18—C19	1.4244 (18)
N2—C22	1.3219 (17)	C18—H18A	0.98
N3—C24	1.2954 (16)	C19—C20	1.4399 (19)
N3—N4	1.3883 (16)	C19—H19A	0.98
N4—C25	1.3296 (16)	C20—C24	1.4524 (16)
N5—C22	1.3511 (18)	C21—H21A	0.93
N5—C23	1.446 (2)	C23—H23A	0.96
N5—H2	0.84	C23—H23B	0.96
N6—C25	1.3467 (18)	C23—H23C	0.96
N6—C26	1.4497 (18)	C24—H24A	0.93
N6—H3	0.82	C26—H26A	0.96
C1—C2	1.423 (2)	C26—H26B	0.96
C1—C5	1.423 (2)	C26—H26C	0.96
C1—H1A	0.98	O1—C27	1.421 (2)
C2—C3	1.426 (2)	O1—H1	0.82
C2—H2A	0.98	C27—H27A	0.96
C3—C4	1.423 (2)	C27—H27B	0.96
C3—H3A	0.98	C27—H27C	0.96
C4—C5	1.421 (2)		
			
N3—Zn1—N1	108.57 (4)	C3—C4—Fe1	69.77 (9)
N3—Zn1—S1	126.07 (3)	C5—C4—H4A	126.1
N1—Zn1—S1	86.60 (3)	C3—C4—H4A	126.1
N3—Zn1—S2	86.89 (3)	Fe1—C4—H4A	126.1
N1—Zn1—S2	117.67 (3)	C4—C5—C1	108.33 (12)
S1—Zn1—S2	132.032 (14)	C4—C5—Fe1	69.54 (8)
C9—Fe1—C10	41.61 (5)	C1—C5—Fe1	69.91 (8)
C9—Fe1—C4	105.73 (6)	C4—C5—H5A	125.8
C10—Fe1—C4	124.75 (6)	C1—C5—H5A	125.8
C9—Fe1—C3	123.91 (6)	Fe1—C5—H5A	125.8
C10—Fe1—C3	162.00 (6)	C7—C6—C10	107.67 (11)
C4—Fe1—C3	40.72 (6)	C7—C6—Fe1	69.85 (8)
C9—Fe1—C5	119.31 (6)	C10—C6—Fe1	68.94 (8)
C10—Fe1—C5	107.36 (5)	C7—C6—H6A	126.2
C4—Fe1—C5	40.66 (6)	C10—C6—H6A	126.2
C3—Fe1—C5	68.33 (6)	Fe1—C6—H6A	126.2
C9—Fe1—C2	161.87 (6)	C8—C7—C6	108.46 (12)
C10—Fe1—C2	155.66 (6)	C8—C7—Fe1	69.60 (8)
C4—Fe1—C2	68.57 (6)	C6—C7—Fe1	69.49 (8)
C3—Fe1—C2	40.75 (6)	C8—C7—H7A	125.8
C5—Fe1—C2	68.33 (6)	C6—C7—H7A	125.8
C9—Fe1—C1	155.09 (5)	Fe1—C7—H7A	125.8
C10—Fe1—C1	120.48 (5)	C9—C8—C7	108.25 (11)
C4—Fe1—C1	68.53 (6)	C9—C8—Fe1	68.66 (7)
C3—Fe1—C1	68.43 (6)	C7—C8—Fe1	69.76 (7)
C5—Fe1—C1	40.63 (6)	C9—C8—H8A	125.9
C2—Fe1—C1	40.61 (6)	C7—C8—H8A	125.9
C9—Fe1—C6	69.45 (5)	Fe1—C8—H8A	125.9
C10—Fe1—C6	41.11 (5)	C8—C9—C10	108.02 (12)
C4—Fe1—C6	163.08 (6)	C8—C9—Fe1	70.70 (7)
C3—Fe1—C6	155.29 (6)	C10—C9—Fe1	69.60 (7)
C5—Fe1—C6	126.44 (5)	C8—C9—H9A	126.0
C2—Fe1—C6	120.91 (6)	C10—C9—H9A	126.0
C1—Fe1—C6	108.63 (5)	Fe1—C9—H9A	126.0
C9—Fe1—C8	40.64 (5)	C6—C10—C9	107.57 (11)
C10—Fe1—C8	68.92 (5)	C6—C10—C21	130.49 (11)
C4—Fe1—C8	118.88 (6)	C9—C10—C21	121.79 (12)
C3—Fe1—C8	106.82 (6)	C6—C10—Fe1	69.95 (7)
C5—Fe1—C8	153.92 (6)	C9—C10—Fe1	68.79 (7)
C2—Fe1—C8	125.71 (6)	C21—C10—Fe1	122.99 (9)
C1—Fe1—C8	163.57 (6)	C15—C11—C12	107.79 (13)
C6—Fe1—C8	68.69 (5)	C15—C11—Fe2	69.86 (8)
C9—Fe1—C7	68.74 (6)	C12—C11—Fe2	69.14 (8)
C10—Fe1—C7	68.75 (5)	C15—C11—H11A	126.1
C4—Fe1—C7	154.22 (6)	C12—C11—H11A	126.1
C3—Fe1—C7	120.16 (6)	Fe2—C11—H11A	126.1
C5—Fe1—C7	164.10 (6)	C13—C12—C11	108.15 (12)
C2—Fe1—C7	108.47 (6)	C13—C12—Fe2	69.35 (7)
C1—Fe1—C7	126.92 (6)	C11—C12—Fe2	70.24 (7)
C6—Fe1—C7	40.67 (5)	C13—C12—H12A	125.9
C8—Fe1—C7	40.64 (6)	C11—C12—H12A	125.9
C16—Fe2—C13	152.48 (6)	Fe2—C12—H12A	125.9
C16—Fe2—C12	118.83 (6)	C12—C13—C14	108.08 (12)
C13—Fe2—C12	40.70 (6)	C12—C13—Fe2	69.95 (8)
C16—Fe2—C17	40.98 (5)	C14—C13—Fe2	70.20 (7)
C13—Fe2—C17	116.23 (6)	C12—C13—H13A	126.0
C12—Fe2—C17	103.37 (6)	C14—C13—H13A	126.0
C16—Fe2—C18	69.00 (6)	Fe2—C13—H13A	126.0
C13—Fe2—C18	103.54 (6)	C15—C14—C13	107.72 (12)
C12—Fe2—C18	120.46 (5)	C15—C14—Fe2	70.05 (8)
C17—Fe2—C18	40.85 (6)	C13—C14—Fe2	68.96 (8)
C16—Fe2—C14	165.53 (5)	C15—C14—H14A	126.1
C13—Fe2—C14	40.83 (6)	C13—C14—H14A	126.1
C12—Fe2—C14	68.42 (6)	Fe2—C14—H14A	126.1
C17—Fe2—C14	152.90 (6)	C11—C15—C14	108.26 (11)
C18—Fe2—C14	119.58 (6)	C11—C15—Fe2	69.74 (8)
C16—Fe2—C11	108.36 (6)	C14—C15—Fe2	69.47 (8)
C13—Fe2—C11	68.43 (6)	C11—C15—H15A	125.9
C12—Fe2—C11	40.63 (5)	C14—C15—H15A	125.9
C17—Fe2—C11	123.14 (6)	Fe2—C15—H15A	125.9
C18—Fe2—C11	158.40 (6)	C17—C16—C20	107.85 (13)
C14—Fe2—C11	68.22 (6)	C17—C16—Fe2	69.94 (8)
C16—Fe2—C19	69.21 (6)	C20—C16—Fe2	70.52 (7)
C13—Fe2—C19	123.10 (6)	C17—C16—H16A	126.1
C12—Fe2—C19	158.23 (5)	C20—C16—H16A	126.1
C17—Fe2—C19	68.67 (6)	Fe2—C16—H16A	126.1
C18—Fe2—C19	40.55 (5)	C16—C17—C18	108.08 (11)
C14—Fe2—C19	108.97 (6)	C16—C17—Fe2	69.08 (7)
C11—Fe2—C19	160.30 (5)	C18—C17—Fe2	69.67 (8)
C16—Fe2—C15	127.98 (6)	C16—C17—H17A	126.0
C13—Fe2—C15	68.35 (5)	C18—C17—H17A	126.0
C12—Fe2—C15	68.16 (5)	Fe2—C17—H17A	126.0
C17—Fe2—C15	162.08 (7)	C19—C18—C17	108.59 (12)
C18—Fe2—C15	157.07 (6)	C19—C18—Fe2	70.21 (8)
C14—Fe2—C15	40.48 (6)	C17—C18—Fe2	69.49 (8)
C11—Fe2—C15	40.40 (6)	C19—C18—H18A	125.7
C19—Fe2—C15	124.83 (5)	C17—C18—H18A	125.7
C16—Fe2—C20	41.18 (5)	Fe2—C18—H18A	125.7
C13—Fe2—C20	162.34 (6)	C18—C19—C20	107.80 (12)
C12—Fe2—C20	156.94 (6)	C18—C19—Fe2	69.24 (8)
C17—Fe2—C20	68.72 (5)	C20—C19—Fe2	69.69 (7)
C18—Fe2—C20	68.48 (5)	C18—C19—H19A	126.1
C14—Fe2—C20	128.23 (5)	C20—C19—H19A	126.1
C11—Fe2—C20	124.55 (5)	Fe2—C19—H19A	126.1
C19—Fe2—C20	40.85 (5)	C19—C20—C16	107.66 (11)
C15—Fe2—C20	112.58 (5)	C19—C20—C24	131.07 (12)
C22—S1—Zn1	92.41 (5)	C16—C20—C24	121.27 (12)
C25—S2—Zn1	92.79 (5)	C19—C20—Fe2	69.46 (7)
C21—N1—N2	118.03 (11)	C16—C20—Fe2	68.30 (7)
C21—N1—Zn1	123.60 (9)	C24—C20—Fe2	128.13 (9)
N2—N1—Zn1	118.31 (8)	N1—C21—C10	129.13 (12)
C22—N2—N1	113.19 (11)	N1—C21—H21A	115.4
C24—N3—N4	117.59 (10)	C10—C21—H21A	115.4
C24—N3—Zn1	124.62 (9)	N2—C22—N5	116.96 (12)
N4—N3—Zn1	117.79 (7)	N2—C22—S1	128.13 (10)
C25—N4—N3	113.85 (10)	N5—C22—S1	114.91 (10)
C22—N5—C23	122.50 (13)	N5—C23—H23A	109.5
C22—N5—H2	118.1	N5—C23—H23B	109.5
C23—N5—H2	117.9	H23A—C23—H23B	109.5
C25—N6—C26	123.40 (11)	N5—C23—H23C	109.5
C25—N6—H3	119.7	H23A—C23—H23C	109.5
C26—N6—H3	116.9	H23B—C23—H23C	109.5
C2—C1—C5	107.77 (12)	N3—C24—C20	129.29 (13)
C2—C1—Fe1	69.52 (7)	N3—C24—H24A	115.4
C5—C1—Fe1	69.46 (7)	C20—C24—H24A	115.4
C2—C1—H1A	126.1	N4—C25—N6	116.34 (11)
C5—C1—H1A	126.1	N4—C25—S2	127.90 (11)
Fe1—C1—H1A	126.1	N6—C25—S2	115.74 (9)
C1—C2—C3	108.03 (13)	N6—C26—H26A	109.5
C1—C2—Fe1	69.87 (7)	N6—C26—H26B	109.5
C3—C2—Fe1	69.57 (8)	H26A—C26—H26B	109.5
C1—C2—H2A	126.0	N6—C26—H26C	109.5
C3—C2—H2A	126.0	H26A—C26—H26C	109.5
Fe1—C2—H2A	126.0	H26B—C26—H26C	109.5
C4—C3—C2	108.01 (12)	C27—O1—H1	112.5
C4—C3—Fe1	69.52 (8)	O1—C27—H27A	109.5
C2—C3—Fe1	69.68 (8)	O1—C27—H27B	109.5
C4—C3—H3A	126.0	H27A—C27—H27B	109.5
C2—C3—H3A	126.0	O1—C27—H27C	109.5
Fe1—C3—H3A	126.0	H27A—C27—H27C	109.5
C5—C4—C3	107.86 (13)	H27B—C27—H27C	109.5
C5—C4—Fe1	69.80 (8)		
			
N3—Zn1—S1—C22	119.19 (6)	C16—Fe2—C11—C15	−127.61 (8)
N1—Zn1—S1—C22	8.62 (5)	C13—Fe2—C11—C15	81.51 (9)
S2—Zn1—S1—C22	−116.32 (4)	C12—Fe2—C11—C15	119.22 (12)
N3—Zn1—S2—C25	6.07 (5)	C17—Fe2—C11—C15	−170.46 (8)
N1—Zn1—S2—C25	115.44 (5)	C18—Fe2—C11—C15	153.17 (15)
S1—Zn1—S2—C25	−132.08 (4)	C14—Fe2—C11—C15	37.41 (8)
N3—Zn1—N1—C21	45.06 (11)	C19—Fe2—C11—C15	−48.1 (2)
S1—Zn1—N1—C21	172.09 (10)	C20—Fe2—C11—C15	−84.86 (9)
S2—Zn1—N1—C21	−51.35 (10)	C16—Fe2—C11—C12	113.17 (9)
N3—Zn1—N1—N2	−137.85 (8)	C13—Fe2—C11—C12	−37.71 (9)
S1—Zn1—N1—N2	−10.82 (8)	C17—Fe2—C11—C12	70.32 (10)
S2—Zn1—N1—N2	125.75 (8)	C18—Fe2—C11—C12	34.0 (2)
C21—N1—N2—C22	−175.16 (11)	C14—Fe2—C11—C12	−81.80 (9)
Zn1—N1—N2—C22	7.58 (13)	C19—Fe2—C11—C12	−167.33 (15)
N1—Zn1—N3—C24	53.54 (11)	C15—Fe2—C11—C12	−119.22 (12)
S1—Zn1—N3—C24	−46.08 (11)	C20—Fe2—C11—C12	155.93 (8)
S2—Zn1—N3—C24	171.74 (10)	C15—C11—C12—C13	−0.19 (15)
N1—Zn1—N3—N4	−126.95 (8)	Fe2—C11—C12—C13	59.19 (9)
S1—Zn1—N3—N4	133.42 (7)	C15—C11—C12—Fe2	−59.38 (9)
S2—Zn1—N3—N4	−8.76 (8)	C16—Fe2—C12—C13	155.84 (8)
C24—N3—N4—C25	−172.80 (11)	C17—Fe2—C12—C13	114.85 (9)
Zn1—N3—N4—C25	7.66 (13)	C18—Fe2—C12—C13	74.51 (10)
C9—Fe1—C1—C2	−162.75 (13)	C14—Fe2—C12—C13	−38.02 (8)
C10—Fe1—C1—C2	159.67 (8)	C11—Fe2—C12—C13	−119.29 (12)
C4—Fe1—C1—C2	−81.69 (10)	C19—Fe2—C12—C13	49.22 (19)
C3—Fe1—C1—C2	−37.77 (9)	C15—Fe2—C12—C13	−81.74 (9)
C5—Fe1—C1—C2	−119.19 (12)	C20—Fe2—C12—C13	−178.34 (12)
C6—Fe1—C1—C2	116.07 (9)	C16—Fe2—C12—C11	−84.88 (10)
C8—Fe1—C1—C2	38.3 (2)	C13—Fe2—C12—C11	119.29 (12)
C7—Fe1—C1—C2	74.48 (10)	C17—Fe2—C12—C11	−125.86 (9)
C9—Fe1—C1—C5	−43.56 (17)	C18—Fe2—C12—C11	−166.20 (9)
C10—Fe1—C1—C5	−81.13 (9)	C14—Fe2—C12—C11	81.27 (9)
C4—Fe1—C1—C5	37.50 (9)	C19—Fe2—C12—C11	168.51 (14)
C3—Fe1—C1—C5	81.42 (9)	C15—Fe2—C12—C11	37.55 (9)
C2—Fe1—C1—C5	119.19 (12)	C20—Fe2—C12—C11	−59.05 (17)
C6—Fe1—C1—C5	−124.73 (8)	C11—C12—C13—C14	0.36 (16)
C8—Fe1—C1—C5	157.54 (19)	Fe2—C12—C13—C14	60.10 (9)
C7—Fe1—C1—C5	−166.32 (8)	C11—C12—C13—Fe2	−59.74 (9)
C5—C1—C2—C3	0.18 (16)	C16—Fe2—C13—C12	−50.89 (16)
Fe1—C1—C2—C3	59.32 (10)	C17—Fe2—C13—C12	−79.78 (10)
C5—C1—C2—Fe1	−59.14 (9)	C18—Fe2—C13—C12	−121.30 (9)
C9—Fe1—C2—C1	156.35 (18)	C14—Fe2—C13—C12	118.85 (12)
C10—Fe1—C2—C1	−46.58 (17)	C11—Fe2—C13—C12	37.64 (8)
C4—Fe1—C2—C1	81.58 (9)	C19—Fe2—C13—C12	−160.41 (8)
C3—Fe1—C2—C1	119.23 (12)	C15—Fe2—C13—C12	81.24 (9)
C5—Fe1—C2—C1	37.72 (9)	C20—Fe2—C13—C12	177.86 (15)
C6—Fe1—C2—C1	−82.76 (10)	C16—Fe2—C13—C14	−169.74 (12)
C8—Fe1—C2—C1	−167.52 (9)	C12—Fe2—C13—C14	−118.85 (12)
C7—Fe1—C2—C1	−125.69 (9)	C17—Fe2—C13—C14	161.37 (9)
C9—Fe1—C2—C3	37.1 (2)	C18—Fe2—C13—C14	119.85 (9)
C10—Fe1—C2—C3	−165.81 (12)	C11—Fe2—C13—C14	−81.21 (9)
C4—Fe1—C2—C3	−37.64 (9)	C19—Fe2—C13—C14	80.74 (10)
C5—Fe1—C2—C3	−81.51 (9)	C15—Fe2—C13—C14	−37.61 (8)
C1—Fe1—C2—C3	−119.23 (12)	C20—Fe2—C13—C14	59.0 (2)
C6—Fe1—C2—C3	158.01 (8)	C12—C13—C14—C15	−0.39 (15)
C8—Fe1—C2—C3	73.25 (11)	Fe2—C13—C14—C15	59.55 (9)
C7—Fe1—C2—C3	115.08 (9)	C12—C13—C14—Fe2	−59.95 (10)
C1—C2—C3—C4	−0.35 (16)	C16—Fe2—C14—C15	41.7 (3)
Fe1—C2—C3—C4	59.15 (10)	C13—Fe2—C14—C15	−119.11 (12)
C1—C2—C3—Fe1	−59.5 (1)	C12—Fe2—C14—C15	−81.22 (8)
C9—Fe1—C3—C4	73.72 (10)	C17—Fe2—C14—C15	−158.10 (12)
C10—Fe1—C3—C4	41.6 (2)	C18—Fe2—C14—C15	165.07 (7)
C5—Fe1—C3—C4	−37.85 (8)	C11—Fe2—C14—C15	−37.34 (8)
C2—Fe1—C3—C4	−119.36 (12)	C19—Fe2—C14—C15	121.85 (8)
C1—Fe1—C3—C4	−81.71 (9)	C20—Fe2—C14—C15	80.22 (10)
C6—Fe1—C3—C4	−169.58 (11)	C16—Fe2—C14—C13	160.8 (2)
C8—Fe1—C3—C4	114.97 (9)	C12—Fe2—C14—C13	37.90 (8)
C7—Fe1—C3—C4	157.13 (8)	C17—Fe2—C14—C13	−38.98 (16)
C9—Fe1—C3—C2	−166.92 (8)	C18—Fe2—C14—C13	−75.82 (9)
C10—Fe1—C3—C2	160.91 (15)	C11—Fe2—C14—C13	81.77 (9)
C4—Fe1—C3—C2	119.36 (12)	C19—Fe2—C14—C13	−119.04 (8)
C5—Fe1—C3—C2	81.51 (9)	C15—Fe2—C14—C13	119.11 (12)
C1—Fe1—C3—C2	37.65 (9)	C20—Fe2—C14—C13	−160.67 (8)
C6—Fe1—C3—C2	−50.22 (16)	C12—C11—C15—C14	−0.06 (15)
C8—Fe1—C3—C2	−125.68 (9)	Fe2—C11—C15—C14	−58.98 (9)
C7—Fe1—C3—C2	−83.51 (9)	C12—C11—C15—Fe2	58.93 (9)
C2—C3—C4—C5	0.39 (16)	C13—C14—C15—C11	0.28 (15)
Fe1—C3—C4—C5	59.64 (10)	Fe2—C14—C15—C11	59.15 (9)
C2—C3—C4—Fe1	−59.25 (10)	C13—C14—C15—Fe2	−58.87 (9)
C9—Fe1—C4—C5	116.91 (8)	C16—Fe2—C15—C11	72.51 (9)
C10—Fe1—C4—C5	75.50 (9)	C13—Fe2—C15—C11	−81.73 (8)
C3—Fe1—C4—C5	−118.94 (12)	C12—Fe2—C15—C11	−37.75 (8)
C2—Fe1—C4—C5	−81.26 (9)	C17—Fe2—C15—C11	26.8 (2)
C1—Fe1—C4—C5	−37.48 (8)	C18—Fe2—C15—C11	−154.77 (12)
C6—Fe1—C4—C5	46.0 (2)	C14—Fe2—C15—C11	−119.65 (11)
C8—Fe1—C4—C5	158.76 (8)	C19—Fe2—C15—C11	162.20 (8)
C7—Fe1—C4—C5	−169.53 (11)	C20—Fe2—C15—C11	117.32 (8)
C9—Fe1—C4—C3	−124.14 (8)	C16—Fe2—C15—C14	−167.84 (8)
C10—Fe1—C4—C3	−165.56 (8)	C13—Fe2—C15—C14	37.92 (8)
C5—Fe1—C4—C3	118.94 (12)	C12—Fe2—C15—C14	81.90 (8)
C2—Fe1—C4—C3	37.68 (8)	C17—Fe2—C15—C14	146.47 (16)
C1—Fe1—C4—C3	81.46 (9)	C18—Fe2—C15—C14	−35.11 (16)
C6—Fe1—C4—C3	164.94 (16)	C11—Fe2—C15—C14	119.65 (11)
C8—Fe1—C4—C3	−82.29 (9)	C19—Fe2—C15—C14	−78.15 (9)
C7—Fe1—C4—C3	−50.58 (16)	C20—Fe2—C15—C14	−123.03 (8)
C3—C4—C5—C1	−0.28 (16)	C13—Fe2—C16—C17	−41.36 (17)
Fe1—C4—C5—C1	59.34 (10)	C12—Fe2—C16—C17	−76.64 (10)
C3—C4—C5—Fe1	−59.62 (10)	C18—Fe2—C16—C17	37.47 (9)
C2—C1—C5—C4	0.06 (16)	C14—Fe2—C16—C17	166.4 (2)
Fe1—C1—C5—C4	−59.11 (10)	C11—Fe2—C16—C17	−119.75 (9)
C2—C1—C5—Fe1	59.17 (9)	C19—Fe2—C16—C17	81.02 (9)
C9—Fe1—C5—C4	−79.84 (10)	C15—Fe2—C16—C17	−160.39 (9)
C10—Fe1—C5—C4	−123.55 (9)	C20—Fe2—C16—C17	118.38 (12)
C3—Fe1—C5—C4	37.90 (9)	C13—Fe2—C16—C20	−159.73 (11)
C2—Fe1—C5—C4	81.90 (9)	C12—Fe2—C16—C20	164.98 (8)
C1—Fe1—C5—C4	119.60 (12)	C17—Fe2—C16—C20	−118.38 (12)
C6—Fe1—C5—C4	−164.92 (8)	C18—Fe2—C16—C20	−80.91 (8)
C8—Fe1—C5—C4	−46.17 (16)	C14—Fe2—C16—C20	48.0 (3)
C7—Fe1—C5—C4	163.23 (18)	C11—Fe2—C16—C20	121.88 (8)
C9—Fe1—C5—C1	160.56 (8)	C19—Fe2—C16—C20	−37.36 (8)
C10—Fe1—C5—C1	116.85 (8)	C15—Fe2—C16—C20	81.24 (10)
C4—Fe1—C5—C1	−119.60 (12)	C20—C16—C17—C18	1.70 (15)
C3—Fe1—C5—C1	−81.70 (9)	Fe2—C16—C17—C18	−58.93 (10)
C2—Fe1—C5—C1	−37.70 (8)	C20—C16—C17—Fe2	60.62 (9)
C6—Fe1—C5—C1	75.48 (10)	C13—Fe2—C17—C16	160.10 (8)
C8—Fe1—C5—C1	−165.77 (11)	C12—Fe2—C17—C16	118.83 (9)
C7—Fe1—C5—C1	43.6 (2)	C18—Fe2—C17—C16	−119.73 (11)
C9—Fe1—C6—C7	80.91 (8)	C14—Fe2—C17—C16	−172.60 (11)
C10—Fe1—C6—C7	119.20 (10)	C11—Fe2—C17—C16	79.76 (9)
C4—Fe1—C6—C7	157.18 (17)	C19—Fe2—C17—C16	−82.44 (9)
C3—Fe1—C6—C7	−46.74 (16)	C15—Fe2—C17—C16	59.32 (19)
C5—Fe1—C6—C7	−167.18 (8)	C20—Fe2—C17—C16	−38.44 (8)
C2—Fe1—C6—C7	−82.52 (9)	C16—Fe2—C17—C18	119.73 (11)
C1—Fe1—C6—C7	−125.48 (8)	C13—Fe2—C17—C18	−80.17 (9)
C8—Fe1—C6—C7	37.26 (8)	C12—Fe2—C17—C18	−121.44 (8)
C9—Fe1—C6—C10	−38.29 (7)	C14—Fe2—C17—C18	−52.87 (15)
C4—Fe1—C6—C10	38.0 (2)	C11—Fe2—C17—C18	−160.51 (7)
C3—Fe1—C6—C10	−165.94 (12)	C19—Fe2—C17—C18	37.29 (7)
C5—Fe1—C6—C10	73.62 (9)	C15—Fe2—C17—C18	179.06 (15)
C2—Fe1—C6—C10	158.28 (7)	C20—Fe2—C17—C18	81.29 (8)
C1—Fe1—C6—C10	115.32 (8)	C16—C17—C18—C19	−0.96 (16)
C8—Fe1—C6—C10	−81.94 (8)	Fe2—C17—C18—C19	−59.52 (9)
C7—Fe1—C6—C10	−119.20 (10)	C16—C17—C18—Fe2	58.56 (9)
C10—C6—C7—C8	−0.09 (15)	C16—Fe2—C18—C19	82.17 (9)
Fe1—C6—C7—C8	−58.85 (10)	C13—Fe2—C18—C19	−125.62 (9)
C10—C6—C7—Fe1	58.76 (9)	C12—Fe2—C18—C19	−165.89 (8)
C9—Fe1—C7—C8	37.18 (8)	C17—Fe2—C18—C19	119.76 (11)
C10—Fe1—C7—C8	81.98 (8)	C14—Fe2—C18—C19	−84.92 (9)
C4—Fe1—C7—C8	−44.97 (16)	C11—Fe2—C18—C19	169.15 (14)
C3—Fe1—C7—C8	−80.62 (9)	C15—Fe2—C18—C19	−59.50 (16)
C5—Fe1—C7—C8	160.65 (18)	C20—Fe2—C18—C19	37.83 (8)
C2—Fe1—C7—C8	−123.76 (8)	C16—Fe2—C18—C17	−37.59 (7)
C1—Fe1—C7—C8	−165.16 (8)	C13—Fe2—C18—C17	114.62 (8)
C6—Fe1—C7—C8	119.99 (11)	C12—Fe2—C18—C17	74.35 (9)
C9—Fe1—C7—C6	−82.82 (8)	C14—Fe2—C18—C17	155.32 (7)
C10—Fe1—C7—C6	−38.01 (7)	C11—Fe2—C18—C17	49.39 (18)
C4—Fe1—C7—C6	−164.96 (12)	C19—Fe2—C18—C17	−119.76 (11)
C3—Fe1—C7—C6	159.39 (8)	C15—Fe2—C18—C17	−179.26 (12)
C5—Fe1—C7—C6	40.7 (2)	C20—Fe2—C18—C17	−81.93 (8)
C2—Fe1—C7—C6	116.25 (8)	C17—C18—C19—C20	−0.16 (15)
C1—Fe1—C7—C6	74.85 (9)	Fe2—C18—C19—C20	−59.23 (9)
C8—Fe1—C7—C6	−119.99 (11)	C17—C18—C19—Fe2	59.07 (10)
C6—C7—C8—C9	0.79 (16)	C16—Fe2—C19—C18	−81.61 (9)
Fe1—C7—C8—C9	−57.99 (9)	C13—Fe2—C19—C18	70.62 (10)
C6—C7—C8—Fe1	58.78 (9)	C12—Fe2—C19—C18	34.50 (19)
C10—Fe1—C8—C9	38.63 (8)	C17—Fe2—C19—C18	−37.55 (9)
C4—Fe1—C8—C9	−80.38 (10)	C14—Fe2—C19—C18	113.65 (9)
C3—Fe1—C8—C9	−122.86 (9)	C11—Fe2—C19—C18	−168.14 (16)
C5—Fe1—C8—C9	−47.92 (16)	C15—Fe2—C19—C18	155.86 (9)
C2—Fe1—C8—C9	−163.63 (9)	C20—Fe2—C19—C18	−119.27 (12)
C1—Fe1—C8—C9	166.54 (18)	C16—Fe2—C19—C20	37.65 (7)
C6—Fe1—C8—C9	82.88 (9)	C13—Fe2—C19—C20	−170.11 (7)
C7—Fe1—C8—C9	120.17 (12)	C12—Fe2—C19—C20	153.77 (14)
C9—Fe1—C8—C7	−120.17 (12)	C17—Fe2—C19—C20	81.71 (8)
C10—Fe1—C8—C7	−81.53 (8)	C18—Fe2—C19—C20	119.27 (12)
C4—Fe1—C8—C7	159.45 (8)	C14—Fe2—C19—C20	−127.08 (8)
C3—Fe1—C8—C7	116.97 (9)	C11—Fe2—C19—C20	−48.9 (2)
C5—Fe1—C8—C7	−168.09 (11)	C15—Fe2—C19—C20	−84.87 (9)
C2—Fe1—C8—C7	76.20 (10)	C18—C19—C20—C16	1.20 (14)
C1—Fe1—C8—C7	46.4 (2)	Fe2—C19—C20—C16	−57.75 (9)
C6—Fe1—C8—C7	−37.29 (8)	C18—C19—C20—C24	−177.82 (13)
C7—C8—C9—C10	−1.17 (15)	Fe2—C19—C20—C24	123.23 (14)
Fe1—C8—C9—C10	−59.84 (9)	C18—C19—C20—Fe2	58.95 (9)
C7—C8—C9—Fe1	58.67 (9)	C17—C16—C20—C19	−1.79 (14)
C10—Fe1—C9—C8	−118.69 (12)	Fe2—C16—C20—C19	58.47 (9)
C4—Fe1—C9—C8	116.24 (9)	C17—C16—C20—C24	177.35 (11)
C3—Fe1—C9—C8	75.66 (10)	Fe2—C16—C20—C24	−122.40 (12)
C5—Fe1—C9—C8	158.02 (8)	C17—C16—C20—Fe2	−60.26 (9)
C2—Fe1—C9—C8	47.3 (2)	C16—Fe2—C20—C19	−119.85 (11)
C1—Fe1—C9—C8	−171.00 (13)	C13—Fe2—C20—C19	28.3 (2)
C6—Fe1—C9—C8	−80.84 (9)	C12—Fe2—C20—C19	−155.26 (13)
C7—Fe1—C9—C8	−37.17 (8)	C17—Fe2—C20—C19	−81.59 (9)
C4—Fe1—C9—C10	−125.08 (8)	C18—Fe2—C20—C19	−37.57 (8)
C3—Fe1—C9—C10	−165.66 (8)	C14—Fe2—C20—C19	73.83 (9)
C5—Fe1—C9—C10	−83.29 (9)	C11—Fe2—C20—C19	162.05 (8)
C2—Fe1—C9—C10	166.01 (17)	C15—Fe2—C20—C19	117.69 (8)
C1—Fe1—C9—C10	−52.32 (17)	C13—Fe2—C20—C16	148.16 (17)
C6—Fe1—C9—C10	37.85 (8)	C12—Fe2—C20—C16	−35.41 (17)
C8—Fe1—C9—C10	118.69 (12)	C17—Fe2—C20—C16	38.26 (9)
C7—Fe1—C9—C10	81.52 (8)	C18—Fe2—C20—C16	82.28 (9)
C7—C6—C10—C9	−0.62 (15)	C14—Fe2—C20—C16	−166.32 (8)
Fe1—C6—C10—C9	58.70 (9)	C11—Fe2—C20—C16	−78.11 (9)
C7—C6—C10—C21	−176.10 (13)	C19—Fe2—C20—C16	119.85 (11)
Fe1—C6—C10—C21	−116.78 (14)	C15—Fe2—C20—C16	−122.46 (8)
C7—C6—C10—Fe1	−59.32 (9)	C16—Fe2—C20—C24	113.45 (16)
C8—C9—C10—C6	1.11 (15)	C13—Fe2—C20—C24	−98.4 (2)
Fe1—C9—C10—C6	−59.43 (9)	C12—Fe2—C20—C24	78.03 (18)
C8—C9—C10—C21	177.07 (12)	C17—Fe2—C20—C24	151.71 (14)
Fe1—C9—C10—C21	116.53 (12)	C18—Fe2—C20—C24	−164.27 (14)
C8—C9—C10—Fe1	60.54 (9)	C14—Fe2—C20—C24	−52.88 (15)
C9—Fe1—C10—C6	119.10 (11)	C11—Fe2—C20—C24	35.34 (15)
C4—Fe1—C10—C6	−167.41 (8)	C19—Fe2—C20—C24	−126.71 (15)
C3—Fe1—C10—C6	160.81 (16)	C15—Fe2—C20—C24	−9.02 (14)
C5—Fe1—C10—C6	−126.04 (8)	N2—N1—C21—C10	1.94 (19)
C2—Fe1—C10—C6	−50.38 (16)	Zn1—N1—C21—C10	179.04 (10)
C1—Fe1—C10—C6	−83.65 (9)	C6—C10—C21—N1	−20.7 (2)
C8—Fe1—C10—C6	81.34 (8)	C9—C10—C21—N1	164.32 (13)
C7—Fe1—C10—C6	37.62 (7)	Fe1—C10—C21—N1	−111.71 (14)
C4—Fe1—C10—C9	73.49 (9)	N1—N2—C22—N5	−177.47 (11)
C3—Fe1—C10—C9	41.7 (2)	N1—N2—C22—S1	2.54 (16)
C5—Fe1—C10—C9	114.86 (8)	C23—N5—C22—N2	9.22 (19)
C2—Fe1—C10—C9	−169.48 (13)	C23—N5—C22—S1	−170.79 (11)
C1—Fe1—C10—C9	157.25 (8)	Zn1—S1—C22—N2	−9.21 (12)
C6—Fe1—C10—C9	−119.10 (11)	Zn1—S1—C22—N5	170.80 (10)
C8—Fe1—C10—C9	−37.76 (8)	N4—N3—C24—C20	−0.55 (19)
C7—Fe1—C10—C9	−81.48 (8)	Zn1—N3—C24—C20	178.95 (10)
C9—Fe1—C10—C21	−114.94 (14)	C19—C20—C24—N3	21.9 (2)
C4—Fe1—C10—C21	−41.45 (12)	C16—C20—C24—N3	−156.98 (13)
C3—Fe1—C10—C21	−73.2 (2)	Fe2—C20—C24—N3	117.22 (14)
C5—Fe1—C10—C21	−0.08 (12)	N3—N4—C25—N6	177.34 (10)
C2—Fe1—C10—C21	75.58 (18)	N3—N4—C25—S2	−0.90 (16)
C1—Fe1—C10—C21	42.31 (12)	C26—N6—C25—N4	−2.80 (18)
C6—Fe1—C10—C21	125.96 (13)	C26—N6—C25—S2	175.66 (10)
C8—Fe1—C10—C21	−152.71 (12)	Zn1—S2—C25—N4	−4.92 (11)
C7—Fe1—C10—C21	163.57 (12)	Zn1—S2—C25—N6	176.83 (9)

### Hydrogen-bond geometry (Å, °)

**Table:**

*D*—H···*A*	*D*—H	H···*A*	*D*···*A*	*D*—H···*A*
O1—H1···N4^i^	0.82	2.13	2.9460 (16)	178
N5—H2···O1^ii^	0.84	2.06	2.8714 (17)	162
C18—H18A···O1^iii^	0.98	2.49	3.4065 (19)	156
C1—H1A···Cg3^iv^	0.98	2.84	3.6176 (15)	137
C11—H11A···Cg1^v^	0.98	2.58	3.4280 (15)	145
C12—H12A···Cg5^iii^	0.98	2.68	3.6607 (14)	175
C27—H27A···Cg4^i^	0.96	2.79	3.3830 (18)	121

Symmetry codes: (i) −*x*+1, −*y*+1, −*z*+2; (ii) *x*, *y*+1, *z*; (iii) *x*+1, *y*, *z*; (iv) *x*−1, *y*, *z*; (v) −*x*+1, *y*+1/2, −*z*+3/2.
